# Regulation of dietary polyphenols on cancer cell pyroptosis and the tumor immune microenvironment

**DOI:** 10.3389/fnut.2022.974896

**Published:** 2022-08-25

**Authors:** Xiaoxia Huang, Yao Wang, Wenhui Yang, Jing Dong, Lin Li

**Affiliations:** ^1^College of Animal Science and Veterinary Medicine, Shenyang Agricultural University, Shenyang, China; ^2^Key Laboratory of Livestock Infectious Diseases, Ministry of Education, Shenyang Agricultural University, Shenyang, China

**Keywords:** tumor immune microenvironment, dietary polyphenols, pyroptosis, curcumin, antitumor immunity

## Abstract

Cancer is a major public health problem that threatens human life worldwide. In recent years, immunotherapy has made great progress in both clinical and laboratory research. But the high heterogeneity and dynamics of tumors makes immunotherapy not suitable for all cancers. Dietary polyphenols have attracted researchers' attention due to their ability to induce cancer cell pyroptosis and to regulate the tumor immune microenvironment (TIME). This review expounds the regulation of dietary polyphenols and their new forms on cancer cell pyroptosis and the TIME. These dietary polyphenols include curcumin (CUR), resveratrol (RES), epigallocatechin gallate (EGCG), apigenin, triptolide (TPL), kaempferol, genistein and moscatilin. New forms of dietary polyphenols refer to their synthetic analogs and nano-delivery, liposomes. Studies in the past decade are included. The result shows that dietary polyphenols induce pyroptosis in breast cancer cells, liver cancer cells, oral squamous cells, carcinoma cells, and other cancer cells through different pathways. Moreover, dietary polyphenols exhibit great potential in the TIME regulation by modulating the programmed cell death protein 1(PD-1)/programmed death-ligand 1 (PD-L1) axis, enhancing antitumor immune cells, weakening the function and activity of immunosuppressive cells, and targeting tumor-associated macrophages (TAMs) to reduce their tumor infiltration and promote their polarization toward the M1 type. Dietary polyphenols are also used with radiotherapy and chemotherapy to improve antitumor immunity and shape a beneficial TIME. In conclusion, dietary polyphenols induce cancer cell pyroptosis and regulate the TIME, providing new ideas for safer cancer cures.

## Introduction

The incidence of cancer has continually risen to 25% since the 20th century. The ever-increasing incidence of cancer is associated with increased production patterns, lifestyles, and life expectancy (1). According to the latest projections from the American Cancer Bureau, 1,918,030 people will be diagnosed with cancer every day in the United States in 2022. In the end, 609,630 people will die of cancer (2). Going back to the nature of cancer itself, cancer can originate in any organ and structure of the body. Unlike other cells, cancer cells can continue to proliferate, replicate indefinitely, and resist death. Even cancer cells can lure immune cells to escape immune evasion (3). Therefore, it is especially difficult to cure cancer. As the three most important treatments, surgery, radiotherapy and chemotherapy have long been and will continue to be the weapons in the fight against cancer. However, all three methods have their own shortcomings and limitations. There is an urgent need to develop more effective anticancer approaches.

Cancer treatment has made landmark achievements in the past decade. In fact, cancer is not a direct cause of death. The weakened immunity and complications of cancer are. Immunotherapy aims to promote the “rebuilding” of the immune system. Some cancer-fighting immune cells are engineered to fight cancer. This involves a new concept of the TIME. In short, the TIME refers to the microenvironment formed by the interaction of tumor cells and immune cells. Some immune checkpoints and cytokines are also included. The TIME is the foundation of immunotherapy. In recent years, research related to immunotherapy has grown exponentially. Clinically, dendritic cells (DC) vaccines, CAR-T cell therapy, adoptive cell transfer, and immune checkpoint inhibitors have achieved surprising results (4).

Immunotherapy is not perfect due to tumor heterogeneity and dynamics. Therefore, it is particularly important to deepen the understanding of the TIME and to find drugs to enhance anti-tumor immunity. In view of this situation, dietary polyphenols have attracted the attention of some researchers due to their superior anti-inflammatory, anticancer, and immunomodulatory functions. Moreover, dietary polyphenols are widely present in everyday foods. They are safe and easily available.

Pyroptosis is a type of programmed cell death. Pro-inflammatory is the most significant feature of pyroptosis that distinguishes it from other programmed cell death such as apoptosis and autophagy (5). Is it possible to artificially induce cancer cell pyroptosis to make cancer regress? This issue is widely discussed. In the study, it is surprisingly found that dietary polyphenols not only induce cancer cell pyroptosis, but also drive a favorable anticancer immunity in the TIME.

## Pyroptosis

**Figure 1:**
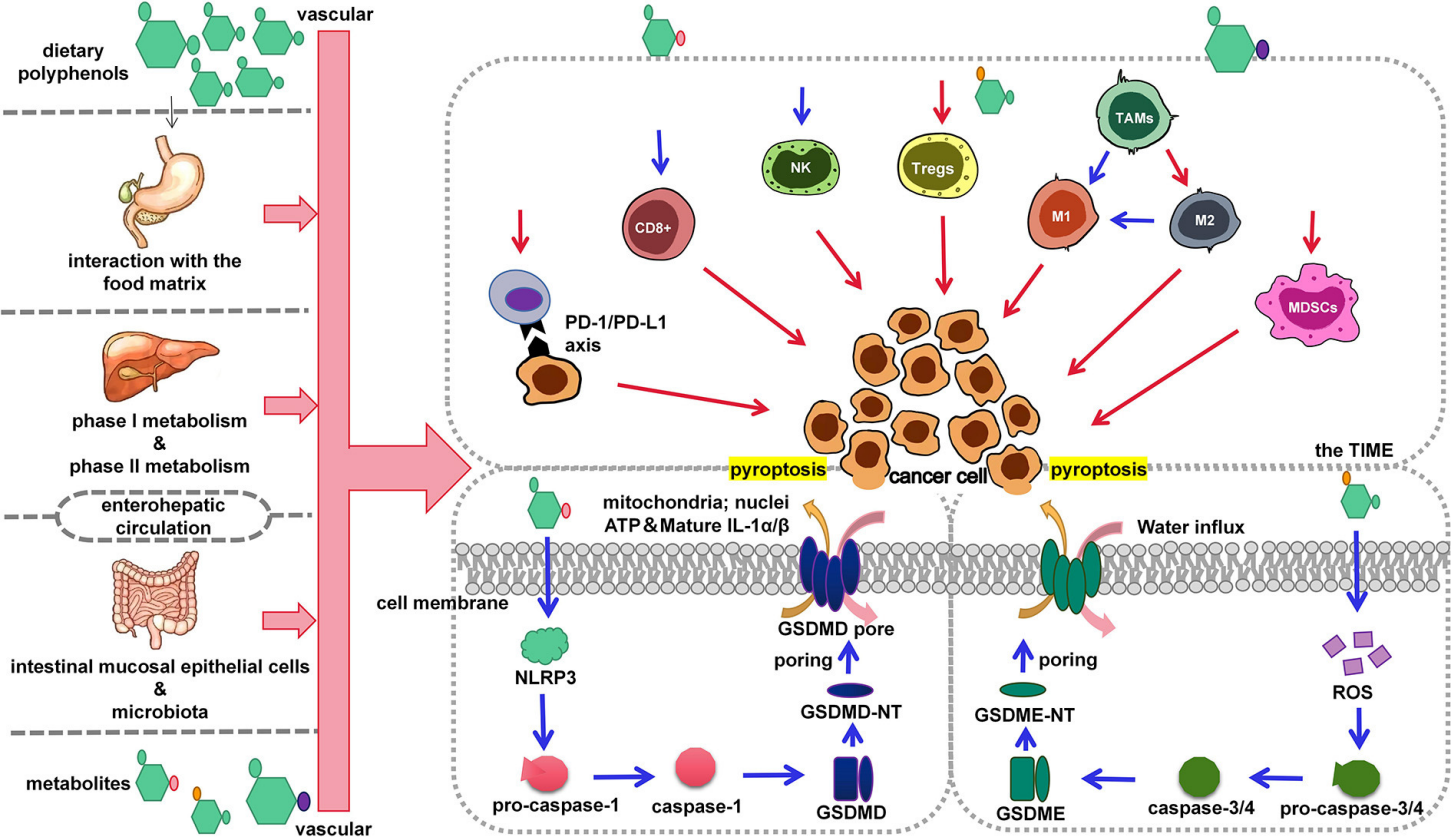
Regulation of dietary polyphenols on cancer cell pyroptosis and the TIME. Dietary polyphenols are ingested orally and interact with food in the stomach. Subsequently, dietary polyphenols undergo phase I and phase II metabolism in the liver. In the gut, dietary polyphenols are metabolized and absorbed by intestinal epithelial cells and gut microbes. The absorbed and digested dietary polyphenols then travel with the blood to the TIME. Immune cells in the TIME are influenced by dietary polyphenols. At the same time, cancer cell pyroptosis was activated by dietary polyphenols. The blue arrow represents promotion and the red arrow represents inhibition.

Pyroptosis is an inflammatory programmed cell death performed by a gasdermin (GSDM) protein family. When pyroptosis is activated, GSDM is cleaved into an auto-inhibitory C-terminal and an active N-terminal (NT). GSDM-NT then punches holes in the cell membrane, causing the cell to swell until it bursts. And a large amount of cellular contents, such as pro-inflammatory factors and lysosomes, are released, resulting in an inflammatory cascade (see Figure 1).

GSDM protein family includes GSDMA, GSDMB, GSDMC, GSDMD, and GSDME. In cancer, GSDMD and GSDME are the most studied. GSDMD induces pyroptosis mainly through two pathways (6). In the classical pyroptosis pathway, inflammasomes recognize pathogen-associated molecular patterns (PAMPs) or damage associated molecular pattern molecules (DAMPs). Then caspase-1 is activated and upregulated, and cleaves GSDMD into GSDMD-NT. This is the first pyroptosis pathway to be studied. In the non-canonical pyroptotic pathway, GSDMD cleavage is performed by caspase-4, 5 (human) and caspase-11 (mouse). Like GSDMD, GSDME also has two pathways. Typically, chemotherapeutic drugs induce caspase-3 to cleave GSDME to perform pyroptosis (7). Recently granzyme B (GZMB) was found to cleave GSDME at D270 to induce pyroptosis instead of caspase-3 (8).

Each GSDM protein has a high degree of expression variability and tissue specificity (8). This is rare in mammals. In different cancers, GSDMD and GSDME have different expression levels and functions. In gastric cancer cells, GSDMD is underexpressed and promotes proliferation (9). In non-small cell carcinoma, GSDMD is highly expressed and indicates higher invasiveness (10). In human colorectal cancer, GSDMD is underexpressed, which is detrimental to patient survival (11). In addition, high expression of GSDMD is associated with poor prognosis of lung adenocarcinoma and osteosarcoma (12). It can be seen that the expression and role of GSDMD in cancer are complex and variable. Even the subcellular localization of GSDMD affects cancer progression and immune response (13). Therefore, the idea of targeting GSDMD to induce cancer cell pyroptosis requires more careful selection and more in-depth exploration.

Different from GSDMD, GSDME acts more as a tumor suppressor. GSDME is normally expressed in the heart, kidney and brain. In most cancers, epigenetics and mutations lead to silencing of the GSDME gene (14). Different expression levels of GSDME determine whether cancer cells tend to apoptosis or pyroptosis during chemotherapy. In cancer cells with high expression of GSDME, caspase-3 specifically cleaves GSDME to convert apoptosis into pyroptosis (7, 15). However, caspase-3 tends to induce apoptosis in cancer cells with low GSDME expression. This phenomenon provides a new idea for anti-apoptotic cancer therapy. In addition to inhibiting cancer cell proliferation, GSDME promotes immune cell infiltration (16, 17). Its expression in tumors converts immunologically “cold” tumors into “hot” tumors, thereby activating antitumor immunity (18).

GSDMA, GSDMB, GSDMC are less studied in cancer. But that doesn't mean they are not important (see Table 1). Using drugs to induce pyroptosis of cancer cells is an important research direction at present. Various studies have shown that pyroptosis has broad prospects in cancer therapy.

**Table 1:** GSDMs and their pathway of pyroptosis.

**GSDMs**	**Pathway**	**References**
GSDMB	CTLs/GZMA/GSDMB	(19)
GSDMC	Caspase-8/GSDMC	(20)
GSDMD	PAMPs or DAMPs/caspase-1/GSDMD	(6)
	LPS/caspase-4, 5, 11/GSDMD	
GSDME	Chemotherapy drugs/caspase-3/GSDME	(7, 8)
	CTLs/GZMB/GSDME	

## TIME

**Figure 2:**
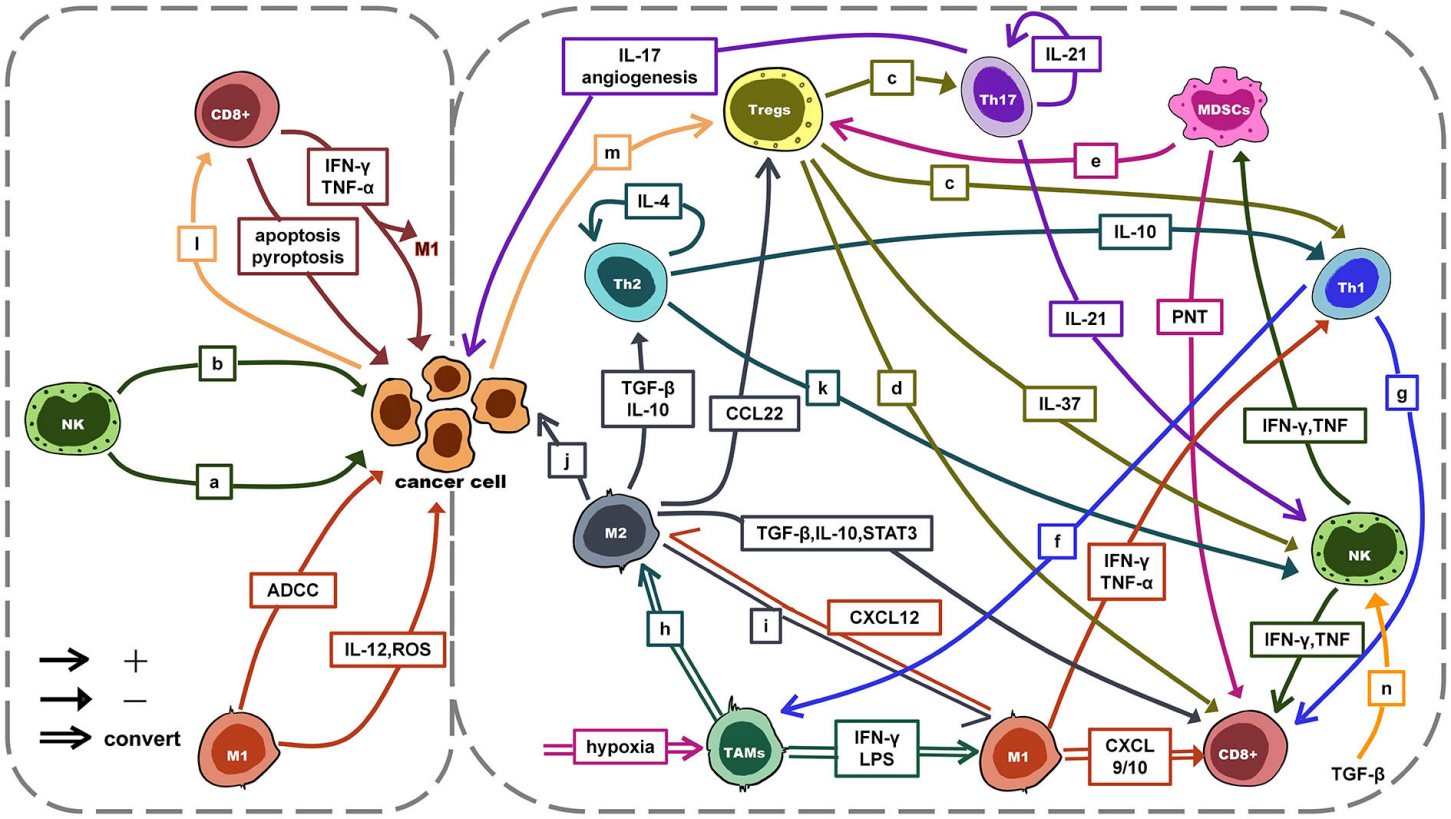
The interaction between antitumor cells, immunosuppressive cells and tumor cells. a: ADCC/apoptosis/pyroptosis; b: IFN-γ, TNF-α: anti-proliferative, anti-angiogenic, pro-apoptosis; c: TGF-β, IL-10, IL-35, GZMB, perforin; d: TGF-β, IL-10, IL-35, GZMB, perforin, CTLA-4, PD-1; e: PD-L1↑, HIF-1α↑, TGF-β, IL-10, CCL4, CCL5; f: IFN-γ: induce activation; g: IFN-γ, LTα, IL-2; h: M2a: IL-4, IL-13; M2b: immune complex with IL-1β/LPS; M2c: IL-10, TGF-β; IL-6, LIF; i: dual blockade of PI3K-γ pathway and CSF-1/CSF-1R; CD40 agonist; j: pro-proliferative, pro-invasion, pro-metastasis, angiogenesis; k: IL-4 → IFN-γ↓; l: MHCI↓ → escape; m: PD-1/PD-L1 → hijack; miR-214 → proliferation; n: cytokine↓; degranulation↓; metabolism↓; mTOR signal↓. ADCC, antibody-dependent cell-mediated cytotoxicity; TGF-β, transforming growth factor-β; CTLA-4, cytotoxic T lymphocyte-associated antigen-4; LIF, leukemia inhibitory; CSF-1, colony-stimulating factor 1 factor; mTOR, mammalian target of rapamycin.

The TIME is a complex ecosystem that acts as a double-edged sword in the progression of cancer. On the one hand, antitumor cells such as NK cells and cytotoxic T lymphocytes (CTLs, mainly CD8+ T cells) can identify and eliminate cancer cells. They play a role in cancer immune monitoring (21–23). On the other hand, immunosuppressive cells such as regulatory T cells (Tregs), TAMs, and myeloid-derived suppressor cells (MDSCs) protect cancer cells by evading immune surveillance. Subsequently, cancer cells can continue to invade, metastasize and induce angiogenesis (21–23). At the same time, cancer cells can induce, expand and recruit a large number of tumor-promoting myeloid cells to establish a tumor immunosuppressive microenvironment by driving immunosuppression, regulating the generation of immune cell subtypes (21, 22). With the joint efforts of anti-tumor immune cells and cancer cells, cancer develops to malignant. However, the role of immune cells in the TIME is not immutable. For example, TAMs, signals in the TIME can directly affect the differentiation of TAMs and polarization between M1 and M2. The interaction between antitumor cells, immunosuppressive cells, and tumor cells is mainly achieved through exosomes, chemokines, and cytokines (see Figure 2) (24–31). This interaction plays a key role in the development of cancer.

All kinds of evidence show that pyroptosis is closely related to the TIME. For example, GSDMB/E can induce pyroptosis after specific cleavage by GZM secreted by CTLs. It was also recently found that macrophage-derived TNF-α activates caspase-8 to cleave GSDMC, resulting in cancer cell pyroptosis (20). However, the role of pyroptosis in the TIME is ambiguous. On the one hand, inflammatory factors released accompanying cancer cell pyroptosis form a chronic inflammatory microenvironment, including NLRP3, IL-18 and IL-1β. This chronic inflammatory microenvironment has been shown to help cancer cells evade innate immune responses and promote cancer progression (10, 32–36). On the other hand, pyroptosis triggers strong antitumor immunity. Pyroptosis significantly increases the accumulation of immune cells and immune factors in solid tumors (37–39). Targeting pyroptosis and stimulating the TIME is a new idea for cancer treatment (40, 41).

The PD-1/PD-L1 axis is a core immunosuppression pathway in the TIME. For a long time, the PD-1/PD-L1 axis has been widely studied due to its immune checkpoint function. However, non-immune checkpoint functions of the PD-1/PD-L1 axis have been identified in studies of pyroptosis. Antibiotic chemotherapeutics induce GSDMC-mediated pyroptosis in hypoxic tumor environments by upregulating the nPD-L1/pro-signal transducer and activator of transcription 3 (p-STAT3) complex (20). In addition, the inflammatory environment created by pyroptosis may enhance the efficacy of anti-PD-L1 therapy. Whether pyroptosis have adverse effects on the immunity of cancer patients? No clear conclusions have been drawn. Is there a substance that can induce cancer cell pyroptosis, and promote anti-tumor immunity at the same time? Dietary polyphenols caught our eye.

## Dietary polyphenols

Polyphenols, which literally means “having multiple phenolic groups,” are mostly found in plant foods. They are widely found in the daily human diet, including nuts, vegetables, fruits, dark chocolate, tea, red wine, and some natural Chinese herbal medicines. They are called “the seventh type of nutrient.” Most natural dietary polyphenols exist in the form of glycoside esters or free aglycones and are biotransformed mainly in the gastrointestinal tract, liver, and gut microbiota (see Table 2) (42–54). Due to factors such as chemical structure and biometabolic properties of dietary polyphenols, their low bioavailability has become a major factor that limits their efficacy *in vivo* and in clinical trials. Chinese herbal medicines rich in polyphenols have been widely used clinically. For example, turmeric, Polygonum cuspidatum, and Tripterygium wilfordii are used to treat cardiovascular disease, rheumatoid arthritis, and systemic lupus erythematosus (55, 56). In recent years, dietary polyphenols have been proven to have a wide range of biological activities. And their effective anti-inflammatory, antioxidant, anticancer, immunomodulatory, and cardiovascular protective properties have made them widely studied and used in food additives, skin care, medicine, health care (57). The anticancer properties of dietary polyphenols are mainly manifested in inhibiting tumor development (proliferation, growth, invasion, metastasis, and angiogenesis), regulating programmed cell death (apoptosis and pyroptosis), inhibiting chemoresistance, enhancing anticancer immune response, and regulating the TIME. In this review, we focus on the properties of dietary polyphenols that induce pyroptosis and regulate the TIME in cancer.

**Table 2:** Dietary polyphenols involved in this review and their names, chemical formulas, structural formulas and metabolic absorption.

**Name**	**Molecular formula**	**Structural formula**	**Primary source**	**Absorption site**	**Metabolic site**	**Metabolites (Enzymes)**	**References**
RES	C_14_H_12_O_3_	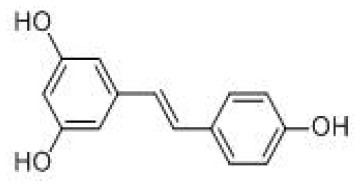	Nuts, grapes, apples, hops, red Fruits, black olives, capers, red rice, red wine, peanuts, berries	Small intestine	Gut	Trans-resveratrol-3-O-sulfate (SULT1A1) Trans-resveratrol-4'-O-sulfate (SULT1A2) Trans-resveratrol-3,4'-O-disulfate (SULT1A2; SULT1A3) Trans-resveratrol-3-O-glucuronide (UGT1A1; UGT1A9) Trans-resveratrol-4'-O-glucuronide (UGT1A1; UGT1A9) Dihydroresveratrol/DHR 3,4'-O-dihydroxy-trans-stilbene Lunularin	(42)
Apigenin	C_15_H_10_O_5_	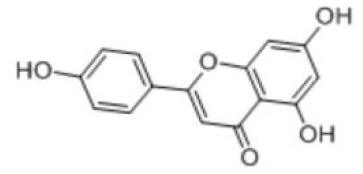	Celery, parsley, peas, chamomile, belimbi fruit, goji leaves	From stomach to colon	Liver; intestine	Glucuronidated apigenin (Phase II Enzymes) Sulphated apigenin (Phase II Enzymes) Luteolin	(43, 44)
CUR	C_21_H_20_O_6_	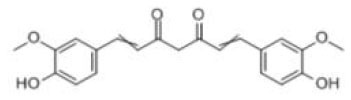	Turmeric, curcuma, calamus	Intestinal lumen, liver		Curcumin glucuronides (UGT1A1;UGT1A8;UGT1A10) Curcumin sulfates (SULT1A1;SULT1A3) Hexahydrocurcumin; Tetrahydrocurcumin (CYP450)	(45, 46)
Anthocyanin		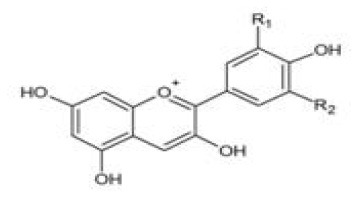 R_1_/R_2_=H,OH,OCH_3_	Blue, purple, and red fruits, flowers, leaves	From stomach to jejunum	Small intestine; big intestine; liver	Anthocyanin glucuronides Anthocyanin methylates Phenolic acid Phenolic acid conjugates	(47)
EGCG	C_22_H_18_O_11_	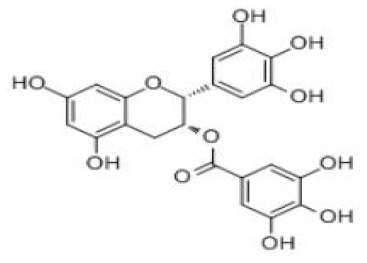	Green tea	Intestine	Gut microbiota; liver	EGC Gallic acid 5-(3,5-dihydroxyphenyl)-4-hydroxyvaleric acid (3,5-dihydroxyphenyl)-γ-valerolactone (-)-5-(5'-hydroxyphenyl)-(4R)- γ-valerolactone 3'-O-β-glucuronide (UGT1A1, 1A8 and 1A9) 4'-O-methyl-EGCG (COMT) 4”-O-methyl-EGCG (COMT) 4-4”-di-O-methyl-EGCG (COMT)	(48, 49)
Quercetin	C_15_H_10_O_7_	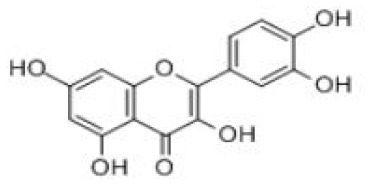	Onions, apples, tea, red wine, edible portion	Small intestine	Liver enteric bacteria; intestinal mucosal epithelial cells; colon bacteria	Thmethylated quercetin Quercetin sulfate Quercetin glucuronidate Phenolic acid Smaller phenolics	(50)
TPL	C_20_H_24_O_6_	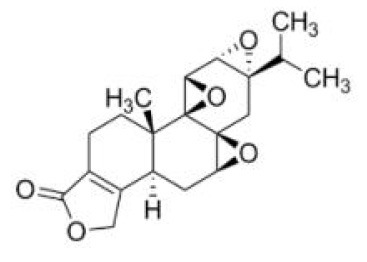	Tripterygium wilfordii *Hook. f*.		Liver microsomes	M1:17-Hydroxytriptolide M2:16-Hydroxytriptolide M3:tripdiolide M4:15-Hydroxytriptolide (CYP3A)	(51, 52)
Genistein	C_15_H_10_O_5_	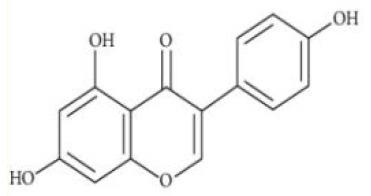	Alfalfa, clover sprouts, broccoli, cauliflower, sunflower, barley meal, caraway, and clover seeds	Small intestine	Liver; small intestine; colon bacteria	Glucuronides (UGT1A8, 1A9, 1A10, 1A1) Sulfates (SULT1A1, 1A2, 1E, 2A1) 3'-OH-Gen, 6-OH-Gen, 8-OH-Gen (CYP1A2, CYP2E1, CYP2D6,CYP3A4) Dihydrogenistein	(53)
Kaempferol	C_15_H_10_O_6_	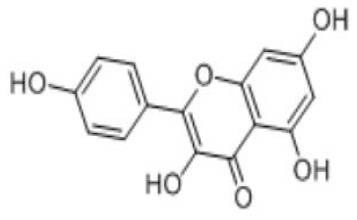	Spinach, kale, dill, chives, tarragon	Small intestine	Liver	Kaempferol-3-glucuronide Kaempferol mono- and di-sulfates	(54)

## Regulation of various dietary polyphenols on cancer cell pyroptosis

Clinically, insufficient intake of dietary polyphenols does not cause a certain disease. However, dietary polyphenol intake is positively correlated with body health (58). As the study of pyroptosis becomes more and more extensive, the inner link between pyroptosis and disease is being revealed. It was found that dietary polyphenols have different effects on pyroptosis in different diseases. Microglia are phagocytic cells that reside in the brain. They play an important role in the immune response after central nervous system injury. Studies have shown that dietary polyphenols protect microglia by inhibiting pyroptosis. This is beneficial for Parkinson's patients and those with spinal cord injuries (59–61). In addition, dietary polyphenols can reduce cardiomyocyte pyroptosis caused by chemotherapeutic drugs (doxorubicin) and adverse environment (ischemia/hypoxia) (62, 63). Studies have also reported that dietary polyphenols inhibit the pathological pyroptosis of liver and kidney cell (64, 65). Dietary polyphenols reduce cell pyroptosis induced by toxic heavy metals. It is of great significance for heavy metal exposure-related diseases. Therefore, we can conclude that dietary polyphenols protect the body by resisting pyroptosis. However, in cancer, dietary polyphenols tend to induce cancer cell pyroptosis to promote the body's victory over cancer. In this section, we discuss how dietary polyphenols induce cancer cell pyroptosis (see Figure 1).

### Dietary polyphenols induce GSDMD-mediated pyroptosis of cancer cells

CUR induces pyroptosis in MCF-7 breast cancer cells through the autophagy/cathepsin B (CTSB)/NLRP3/caspase-1/GSDMD signaling pathway (66). Anthocyanin activates pyroptosis of Tca8113 and SCC15 oral squamous cell carcinoma cells through the NLRP3/caspase-1/GSDMD pathway. Besides, anthocyanin inhibits cancer cell viability, invasion, and metastasis (67). Similar to anthocyanin, chrysophanol up-regulated the expression of NLRP3 in gastric cancer cells. Subsequently, the caspase-1/GSDMD pathway is activated to induce cancer cell pyroptosis (68).

### Dietary polyphenols induce GSDME-mediated pyroptosis of cancer cells

By treating different head and neck cancer cells with TPL, it has been found that TPL selectively induced pyroptosis in an HK1 squamous cell carcinoma cell line and a FaDu hypopharyngeal carcinoma cell line. This pyroptosis was achieved by inhibiting the expression of c-myc and mitochondrial hexokinase II (HK-II) in cancer cells, leading to the activation of the BAD/BAX-caspase-3 cascade, and then cleaves GSDME (69). CUR up-regulates ROS levels, down-regulates pro-caspase-3 expression, and up-regulates GSDME-NT expression in a time- and dose-dependent manner to promote pyroptosis in HepG2 hepatoma cells (70). Kaempferol has been shown to trigger GSDME-mediated U87 MG and U251 glioblastoma cell line pyroptosis by inducing high levels of ROS autophagy and activating inflammasome/caspase-1/IL-1β signaling *in vitro* and *in vivo* experiments (71). In another study on glioblastoma, researchers used genomic data to find that human gliomas express higher levels of GSDME than normal brains. Subsequently, *in vivo* and *in vitro* tests were carried out. They found that galangin induces GSDME-mediated pyroptosis in glioblastoma cells (72). Neobractatin is a kind of dietary polyphenol extracted from Garcinia bracteata. In esophageal cancer cells with high GSDME expression, neobractatin induces pyroptosis through the caspase-3/GSDME pathway. After GSDME was knocked out, pyroptosis transformed into apoptosis. Neobractatin treatment of esophageal cancer showed significant tumor regression (73). *Spatholobus suberectus* Dunn is called “Ji Xue Teng” in Chinese. Its percolation extract contains various dietary polyphenols such as catechin, procyanidin B2, epicatechin, genistein, and formononetin. *Spatholobus suberectus* Dunn percolation extract induced pyroptosis of triple-negative breast cancer cells is also mediated by GSDME. However, GSDME is activated by caspase-4 rather than caspase-1 (74).

### Related research on dietary polyphenol-induced pyroptosis of cancer cells

In some studies, dietary polyphenols have been shown to induce cancer cell pyroptosis. But the pyroptosis pathway was not revealed. These findings are also included in this review.

CUR delivers a double whammy against malignant mesothelioma cells. It induces pyroptosis through activation of caspase-1 by ROS. Moreover, CUR significantly down-regulated the expression levels of inflammasome-related genes such as NF-κB, toll-like receptor (TLR), and IL-1β (75). Researchers linked CUR to sound photodynamic therapy (SPDT). *In vitro* experiments showed that HepG2 cells underwent pyroptosis and apoptosis with CUR-PLGA-MB-SPDT (CUR-loaded poly (L-lactide-co-glycolide)-microbubble (MB)-mediated SPDT) treatment. The underlying mechanism is related to the loss of mitochondrial membrane potential (MMP) and the increase of ROS (76).

Moscatilin and RES can act as radiosensitizers in combination with 1 Gy X-ray or 200 J/m2 UV-C radiation. Overlapping cell death pathways were activated by this combination in HepG2, SH-SY5Y and HaCaT cell lines, including necroptosis and pyroptosis (77). Moscatiline induces immunogenic death of cancer cells. The combination of Moscatiline and radiation induces pyroptosis of cancer cells, eventually leading to necroptosis (77). These findings validate the fact that dietary polyphenols induce cancer cell pyroptosis (see Table 3).

**Table 3:** Related studies on dietary polyphenols-induced pyroptosis of cancer cells.

**Dietary polyphenols**	**Experimental model**	**Dose; treatment time**	**Mechanism**	**Pyroptosis pathway**	**References**
CUR	*In vitro*: MCF-7 breast cancer cell line	8 μM; 24 h	↑LC3, CTSB, ASC, pro-caspase-1, GSDMD, NLRP3, caspase-1, GSDMD-N, IL-1β, IL-18 ↓P62	Autophagy/CTSB/NLRP3/ caspase-1/GSDMD	(66)
	*In vivo*: Six-week-old SPF female BALB/c nude mice vaccinated with MCF-7; 5pcs/group, 2 groups	200 μg/kg/d; 4 weeks			
Anthocyanin	*In vitro*: Tca8113 and SCC15 oral squamous cell carcinoma cell lines	250 μg/ml; 48 h	↑NLRP3, caspase-1,GSDMD, IL-1β	NLRP3/ caspase-1/GSDMD	(67)
TPL	*In vitro*: HK1 squamous cell carcinoma cell line *In vitro*: FaDu hypopharyngeal carcinoma cell line	0, 5, 25, 50, 150 nM; 24 h, 48 h	↓HK-II ↑BAD/BAX-caspase-3, GSDME	HK-II/(BAD/ BAX-caspase-3)/GSDME	(69)
	*In vivo*: 5-week-old male BALB/c nude mice	1 mg/kg/d; 10 d			
CUR	*In vitro*: HepG2 human liver cancer cell line	0, 20, 30 μM; 12 h	↓full length GSDME, pro-caspase-3; Bcl-2 ↑GSDME-N, Bax, ROS, LDH	ROS/caspase-3 /GSDME	(70)
Kaempferol	*In vitro*: U87 MG and U251 Glioblastoma cell line	0, 20, 40, 80, 120 μM; 24 h	↑ROS, IL-1β, ASC, P62, caspase-3, GSDME	ROS/caspase-3 /GSDME	(71)
	*In vivo*: 6-week-old male immune-deficient BALB/c nude mice vaccinated with U87 MG	40 mg/kg/2d; 3 weeks			
CUR	*In vitro*: HMESO malignant mesothelioma cells	40 μM; 48h	↓NF-κB, TLR, IL-1β, ASC ↑caspase-1, HMGB1, ROS	—	(75)
	*In vivo*: allograft model: 8 week-old male C57/BL6 mice vaccinated with mouse MM cells, 4-8 pcs/group *In vivo*: xenograft model:6–8 week-old male Fox Chase SCID mice vaccinated with HMESO cells, 4-8pcs/group	—; 3 weeks			
CUR-PLGA-MB-SPDT	*In vitro*: HepG2 human liver cancer cell line	0, 1.25, 2.5, 5, 10, 20, 40, 80 μM, 2/3 h	↑ROS, mitochondrial depolarization	—	(76)
Moscatilin RES	*In vitro*: HepG2, SH-SY5Y, HaCaT cell line	1, 10, 12.5 μg/ml 5 μg/ml moscatilin/resveratrol + X-ray (1 Gy)/UV-C (200 J/m2)	↑cell-cycle arrest, radiosensitivity	—	(77)

In fact, dietary polyphenols induce not only cancer cell pyroptosis but also various regulated cell deaths such as apoptosis, ferroptosis and autophagy (78–80). While one of cell death is inhibited, dietary polyphenols enhance other cell death (72). This is due to the crosstalk between different cell deaths. Therefore, targeting pyroptosis may be a potential therapy for some apoptosis-resistant cancer. And as mentioned in the section 3, pyroptosis may have a positive effect on the TIME. Taken together, targeting cancer cell pyroptosis is undoubtedly important and meaningful. So, we focused on the role of dietary polyphenols on cancer cell pyroptosis in this review. However, it is extremely important that dietary polyphenol-induced cancer cell death is not single.

## Regulation of various dietary polyphenols on the TIME

### Dietary polyphenols shape an advantageous TIME by modulating the PD-1/PD-L1 axis

Among all immune checkpoint inhibition points, the PD-L1/PD-1 axis was the most well-studied. Because of its value as a therapeutic target for patients with multiple malignancies and advanced cancers, the PD-L1/PD-1 axis has received extensive attention. PD-L1 binds to PD-1 on the surface of antigen-specific T cells, and suppress antitumor immunity and maintain self-tolerance by regulating the number and activity of antigen-specific T cells in the TIME (81). Some studies have shown that dietary polyphenols can regulate the PD-1/PD-L1 axis between tumor cells and immune cells, driving the TIME to develop in an antitumor direction.

Studies have found that RES directly destroys the N-linked glycan modification and dimerization of PD-L1 in JIMT-1 breast cancer cells, inhibits the correct localization of glycosylated PD-L1 to the cell membrane, blocks the PD-1/PD-L1 axis between CTLs and cancer cells. The destruction of PD-1/PD-L1 axis by RES reduces the immune escape of cancer cells and enhances the immune activity of CTLs (82). RES upregulated PD-L1 expression in lung cancer cells *in vitro* and significantly induced apoptosis in Jurkat T cells with high PD-1 expression. The mechanism is related to the activation of the canonical Wnt signaling pathway and the reduction of IFN-γ in Jurkat T cells (83). Both luteolin and apigenin can inhibit STAT3 phosphorylation in non-small cell lung cancer (NSCLC) cells, down-regulate IFN-γ-induced PD-L1 expression, increase the activity and function of CD8+ T cells, and enhance the infiltration of CD8+ T cells in tumors (84). Like luteolin and apigenin, CUR can reduce STAT1 phosphorylation and inhibit IFN-γ-induced PD-L1 up-regulation in A375 human melanoma cells *in vitro* (85). For tongue squamous cell carcinoma, CUR down-regulates PD-L1 expression *in vitro* and *in vivo* and inhibits immunosuppressive signaling. CUR also increases CD8 + T cells in immune infiltration, promotes anti-tumor immunity (86).

It is not difficult to see the synergistic effect of dietary polyphenols and anti-PD-1. And this inference has been confirmed in multiple studies. For luteolin and apigenin, anti-PD-1 can amplify the TIME-regulating effect of luteolin and apigenin and significantly enhance the anticancer effect, providing a prospective treatment strategy for KRAS-mutated NSCLC (84). CUR can reduce the expression of TGF-β1 and PD-L1 on the surface of hepatoma cells and inhibit the PD-1/PD-L1 axis both *in vitro* and *in vivo*. At the same time, the proportion of CD8+ T cells increased and the expression of Foxp3+ Tregs decreased, which promoted anti-tumor immunity. Besides, the combination of anti-PD-1 and CUR significantly enhanced the TIME regulation function of CUR (87).

### Bidirectional regulation of the TIME by dietary polyphenols by enhancing antitumor cell effects and attenuating immunosuppressive cell effects

As mentioned in section 2, antitumor cell immunity and immunosuppression in the TIME are in a state of mutual confrontation that is like two sides of a scale. Regardless of the form of action, dietary polyphenols always tip the balance in favor of the antitumor side (see Figure 1). CUR inhibits the maturation and immunosuppressive function of MDSCs by inhibiting the levels of arginase-1 (Arg-1), ROS, and IL-6 in the Lewis lung cancer model, reducing the inhibitory effect of MDSCs on T cell proliferation (88). The proportion of IFN-γ and CD8+ T cells increased and the proportion of CD4+ T cells decreased when huh-7 human hepatoma cells were treated with CUR. The addition of taurine significantly enhanced the effect of CUR (89, 90). In another study, it was found that curcuminoids promoted pancreatic cancer cell apoptosis by enhancing the cytotoxicity of NK cells (90, 91). Subsequent experiments showed that the combination of CUR, omega-3 fatty acids and Smartfish (antioxidant-rich lipid emulsion) further enhanced the toxic effects of NK cells on pancreatic cancer cells. And CUR prevents NK cells from producing IFN-γ (90, 91). The researchers attempted to compare and combine CUR with Poly I: C (Toll-like receptor 3 agonist, PIC). It was demonstrated that CUR effectively inhibited PIC-dependent NF-κB activation and Tregs recruitment in head and neck squamous cell carcinoma, demonstrating a beneficial TIME regulation (92). In the co-culture system of MDA-MB-231 and NK-92, CUR bidirectionally enhanced the anticancer activity of NK-9 cells. CUR increases the expression and proportion of Stat4 and STAT5 in CD16+ and CD56dim NK-9 cells. In addition, the expression of pErk and PI3K in MDA-MB-231 was significantly downregulated by CUR (93). In the A375 mouse xenograft model, apigenin was observed to inhibit melanoma growth by significantly enhancing immune cell infiltration and T-cell-mediated tumor-cell killing (85). By comparison, CUR also has the same TIME-regulating effect as apigenin but is not as significant as that of apigenin (85).

CUR is the most dietary polyphenol in regulating the TIME, but other dietary polyphenols such as RES, EGCG, anthocyanins, and genistein are not inferior. RES enhances the overall cytotoxicity of NK cells, resulting in strong activation of NK cells in the TIME. The addition of IL-2 enhanced the activation of NK cells in tumors (94). When non-cytotoxic concentrations of RES were used to treat hepatocellular carcinoma mice, RES was found to enhance anti-tumor immunity by reducing levels of Tregs and M2 macrophages and up-regulating levels of CD8+ T cells (95). Meanwhile, RES also modulated the TIME by regulating related cytokines, for example, up-regulating the anti-tumor cytokines TNF-α and IFN-γ, and down-regulating the immunosuppressive cytokines TGF-β1 and IL-10 (95). Anthocyanin treatment of N-nitrosomethylbenzylamine-induced esophageal papilloma in rats showed good antitumor properties (96). Anthocyanins significantly reduced the recruitment and infiltration of CD68+/CD163- macrophages and CD163+ macrophages in tumors and reduced the accumulation of neutrophils (96). EGCG increased the proportion of CD4+ Tregs and CD8+ T cells in the breast cancer TIME and enhanced the antitumor immune response (83). In addition, EGCG targeted MDSCs in tumors through the Arg-1/iNOS/Nox2/NF-κB/STAT3 classical pathway and non-classical pathways, such as the PI3K-Akt signaling pathway, focal adhesions, and ECM-receptor interactions, reducing their proportion (97). Genistein significantly reduced immune avoidance marker Foxp3 in tumors and upregulated cytotoxic T cell marker Cd8a at mRNA level. And lifetime intake of genistein improves anti-tumor immune response (98).

### Dietary polyphenols targeting TAMs promote antitumor immunity in the TIME

TAMs affect tumor progression in different ways. They can be roughly divided into two distinct polarization states: antitumor M1 type and pro-tumor immunosuppressive M2 type. In recent years, TAMs targeting strategies have become a hot spot in antitumor therapy. Some dietary polyphenols inhibit the infiltration and polarization of TAMs to M2 type or promote TAMs polarization from M2 type to M1 type in the TIME. Studies found that apigenin restored SHIP-1 expression in pancreatic cancer mice, promoted MDSCs homeostasis, increased M1 expression and M2 polarized to M1, resulting in tumor regression (99). EGCG up-regulates Mir-16 in breast cancer cell exosomes both *in vitro* and *in vivo*. Subsequently, TAMs received mir-16 transferred from exosomes. Finally, TAMs infiltration and M2-type polarization were inhibited to inhibit NF-κB activity through the IKKα/Iκ B/NF-κB pathway (100). Hs-1793, a synthetic RES analog, induces upregulation of IFN-γ in tumor tissue in breast cancer mice, leading to a significant reduction in M2 invasion and reprogramming. Favorable TIME changes were associated, such as decreased infiltration of Tregs and decreased immunosuppressive mediators (101).

### Novel forms of dietary polyphenols enhance their bioavailability and enhance their TIME-regulating activity

Due to low solubility and bioavailability of dietary polyphenols, it is difficult to reach serum concentrations in clinical experimental models. Therefore, dietary polyphenols have certain shortcomings in clinical applications. In recent years, researchers have used liposome coating, nano-delivery systems, and synthetic analogs to improve this problem. The anticancer and the TIME-regulating effects of dietary polyphenols were significantly enhanced.

Cur-loaded nanomicelles (CUR@PPC) showed good TIME-regulating activity against melanoma *in vitro* in two aspects. First, it affects the expression of cytokines, including down-regulation of CCL-22, PD-L1, TGF-β and IL-10, and up-regulation of IFN-γ and TNF-α. Second, it affects immune cell infiltration, including reducing Tregs and enhancing CD8+ T cell immune infiltration (102). This study also found that anti-PD-1 and T cell-delivered NF-κB inhibitors synergized and promoted each other with CUR@PPC, showing good TIME regulation and antitumor effects. This founding provides a new idea for the combined treatment of cancer with dietary polyphenols and immunotherapy (102). In a study of glioblastoma (GBM), researchers found that both liposomal TriCurin (CUR: EGCG: RES = 4:1:12.5) and phytosomal CUR (CCP) therapy induced GBM cells, GBM stem cells apoptosis, and M2-to-M1 polarization (103, 104). In studies using CCP, a large number of activated NK cells were found in the TIME, accompanied by M2-to-M1 polarization. It is speculated that monocyte chemoattractant protein-1 (MCP-1/CCL2) produced by M1 microglia in CCP-induced GBM first acts to induce M1 activation and release IL-12. Subsequently, IL-12 stimulates NK cells to express Cc chemokine receptor 2 and recruit NK cells to the TIME (104). In another experiment using tricurin, the researchers found similar results. Tricurin induces M2 to M1 polarization in human papillomavirus tumors, and IL-12-dependent NK cells and CTLs were recruited to the TIME (105), Nano-curcumin and RES work together to arrest the cell cycle reduce cell viability in rectal cancer cells. *In vivo* experiments, nanocurcumin and RES promoted macrophage recruitment and enhanced T lymphocyte infiltration (106). *In vitro* experiments, nano-curcumin was reported to support antitumor cells against pancreatic cancer through various pathways, including enhancing the expression of CD86 and driving DC maturation; significantly reducing the levels of various pro-inflammatory cytokines, such as TNF-α, IL-8, IL-6, IL-10, and IL-1 in activated T cells; and down-regulating IL-8 and up-regulating IFN-γ expression in CTLs (107, 108). CUR analog GO-Y030 exerts potent anticancer effects by reducing the generation, stability, and secretion (TGF-β, IL-10) of Tregs in the melanoma TIME (109). Trans-Scirpusin A (TSA), a natural oligomer of RES. It reduces the number and ratio of Tregs and MDSCs in mouse colorectal cancer tumor tissue and induces antitumor immunity (108).

### Dietary polyphenols play a role as adjuvants in regulating the TIME to improve the efficacy of chemotherapy and radiotherapy

Although immunotherapy and precision medicine have risen rapidly with the deepening of research in recent years. Surgery, chemotherapy, and radiotherapy are still the most utilized cancer treatments in clinical practice. Some scholars have tried to use dietary polyphenols to alleviate the interference of chemotherapy resistance and radiotherapy on the TIME, and have made some progress.

TPL reduces cisplatin (DDP) resistance in epithelial ovarian cancer mice and synergizes with DDP (110). Both TPL and TPL combined with DDP significantly increased the levels of NK cell-related proteins CD16 and CD56 in the TIME, and promoted cancer cell apoptosis. It provides a new possibility for improving the survival rate of patients with chemotherapy-resistant advanced ovarian cancer (110). Mammary chimeric mice that received sparse ionizing radiation (SIR) and dense ionizing radiation (DIR) had higher tumor incidence and tumor growth rates, accompanied by a distinct tumor immunosuppressive microenvironment (111). It manifested as a lack of lymphocyte infiltration, increased immunosuppressive myeloid cells, a lack of CD8+ T cells in some aged and fast-growing tumor mice, and a high expression of COX-2, PD-L1, and TGF-β (111). However, Phenyl caffeate was reported to effectively reverse this adverse effect of irradiation on the TIME (111). Joong Sun Kim et al. combined HS-1793 with radiotherapy. They found that HS-1793 could effectively alleviate the adverse effects of radiotherapy on the TIME in FM3A tumor-bearing mice by reducing the number and the infiltration of Tregs in tumor tissue. Meanwhile, HS-1793 upregulated the number of CD8+ T cells and upregulated IFN-γ secretion to attenuate TAM-induced immunosuppression (112). In another study, it was also found that modulated electrothermal therapy could create a more favorable TIME and significantly enhance the immunomodulatory and antitumor effects of nanocurcumin and RES (106) (see Supplementary Table 1).

## Conclusions

In this review, we systematically reviewed the effects of dietary polyphenols on cancer cell pyroptosis and the TIME. The results showed that dietary polyphenols induced pyroptosis in cancer cells mainly through the GSDMD and GSDME pathways. Furthermore, dietary polyphenols regulate the TIME by enhancing antitumor immune cells and weakening immunosuppressive cells. At the same time, TAMs are targeted by dietary polyphenols and reduce their tumor infiltration and promote their polarization from M2 to M1 type. Of course, there are also some issues that deserve to be considered.

Elevated IL-18, IL-1β, NLRP3 are one of the main features of pyroptosis. These three inflammatory factors are often used as indicators of pyroptosis. However, overproduction of IL-18/1β and NLRP3 leads to neonatal-onset multisystem inflammatory disease (NOMID) which damages the spleen, skin, liver, and bone a lot. Studies have shown that the pathogenesis of NOMID is GSDMD-dependent (113). The concomitant effects of IL-18, IL-1β and NLRP3 elevation were ignored in studies claiming that GSDMD mediates pyroptosis in cancer cells. The same problem exists in all studies targeting pyroptosis to treat cancer. Some studies of dietary polyphenols inducing pyroptosis in cancer cells were performed only in cells. Unfortunately, in the *in vitro* studies, the effects of pyroptosis on the TIME and inflammation were ignored too. This may affect the prognosis and complications of cancer patients. There is no doubt that dietary polyphenols are beneficial for anti-tumor immunity in patients. But does this conclusion hold when using drugs to induce pyroptosis in cancer? It is a pity that this question has not been explored in depth.

Dietary polyphenols can reduce levels of pro-inflammatory mediators. This conclusion has been verified in many experiments. Taking CUR as an example, CUR alleviates inflammatory diseases by reducing major markers of inflammation, such as IL-6 and TNF-α, malondialdehyde, sensitive C-reactive protein, MCP-1 (114–116). The anti-inflammatory effect of dietary polyphenols is the basis of their biological activity. Are dietary polyphenols beneficial to the release of inflammatory factors induced by pyroptosis in cancer cells? This requires further research and discussion. Perhaps, we should consider the inflammatory nature of pyroptosis, the TIME, and pyroptosis as a whole. The global effects of dietary polyphenols targeting pyroptosis need to be discussed systematically. Accelerate the pace of dietary polyphenols from laboratory to clinical.

There is no doubt that the TIME is a huge, complex system. How to adjust the TIME to make it a tool to fight cancer will be a research hotspot into the future. Deeper research and exploration are required on other pathways and mechanisms of pyroptosis incancer, the role of pyroptosis in tumor development, the impact of cancer cell pyroptosis on the TIME, and the appropriate degree of cancer cell pyroptosis. Among the above issues, dietary polyphenols deserve further attention. Of course, when it comes to dietary polyphenols, their limited bioavailability cannot be ignored. Related researchers have greatly improved the bioavailability of dietary polyphenols by using nano-delivery systems, liposome coating. Dietary polyphenols are one step closer to clinic.

Pyroptosis is a new field. Targeting pyroptosis to treat cancer holds a aboard future. Dietary polyphenols offer a safer option. Based on the current research, it is also a good idea to combine dietary polyphenols with other drugs. Dietary polyphenols are also excellent as adjuvants for chemotherapy and radiotherapy.

## Author contributions

JD and LL conceived and designed the study. XH and YW collected the literature and drafted the manuscript. JD, LL, and WY helped to review and revise the manuscript. All authors read and approved the final paper.

## Funding

This work was supported by the National Natural Science Foundation of China (No. 32102736), Ruipeng Foundation and New Ruipeng Pet Medical Group Co., LTD (No. RPJJ2020033), Liaoning Province Xing Liao Talents Program Project (No. XLYC1807120), Shenyang Young and Middle-aged Scientific and Technological Innovation Talent Support Program (No. RC200431), Liaoning Province High-level Innovation Team Overseas Training Project (No. 2018LNGXJWPY-YB017), and Liaoning Province General Undergraduate Intercollegiate Joint Training Project (No. 2021-24).

## Conflict of interest

The authors declare that the research was conducted in the absence of any commercial or financial relationships that could be construed as a potential conflict of interest.

## Publisher's note

All claims expressed in this article are solely those of the authors and do not necessarily represent those of their affiliated organizations, or those of the publisher, the editors and the reviewers. Any product that may be evaluated in this article, or claim that may be made by its manufacturer, is not guaranteed or endorsed by the publisher.
