# Supplementary material for: Regulation of dietary polyphenols on cancer cell pyroptosis and the tumor immune microenvironment
Source: Front Nutr. 2022 Aug 25;9:974896. doi: 10.3389/fnut.2022.974896 (PMC9453822; doi:10.3389/fnut.2022.974896)
Supplement: Supplementary file 1 [file Table_1.doc]

Table S1 Related studies on dietary polyphenols regulating the TIME

| Dietary polyphenols | Experimental model | Dose; treatment time | Experimental result | References |
| --- | --- | --- | --- | --- |
| RES | in vitro: JIMT-1 breast cancer cell-T cell co-culture system | 100 μg/mL; 24 h | + CTLs activity; abnormal glycosylation modified PD-L1 endoplasmic reticulum retention | (82) |
| RES | in vitro: A549 and H1299 human lung adenocarcinoma cell lines-human Jurkat T cell leukemia cells co-cultivation system | 20 μM; - | + PD-L1 expression in cancer cells  − T cell proliferation and IFN-γ secretion | (83) |
| apigenin  /luteolin | in vitro: H358, H460, H2122, and A549 KRAS-mutant human lung carcinoma cell lines | -; - | + KRAS mutant; CD8+ T cell infiltration in tumor  − PD-L1 expression induced by IFN-γ; phosphorylation of STAT3 | (84) |
|  | in vivo: 8-week-old female nude mice vaccinated with H358 cells | apigenin/luteolin: 30 mg/kg; 1 time/day; ip |  |  |
|  | in vivo: Lewis lung carcinoma model | apigenin/luteolin: 30 mg/kg, 1 time/day and anti-PD-L1 mAb: 10 mg/kg, 2 times/week; ip |  |  |
| CUR  apigenin | in vitro: A375 human melanoma cell lines-Jurkat cells / DC co-cultivation system | pretreated with curcumin:25 μM, 4h  pretreated with apigenin:30 μM ; 4h  A375 with Jurkat cells: 72h  A375 with DC: 7d | + apoptosis;T cell-mediated melanoma cell killing  − proliferation; IFN-γ-induced PD-L1 expression | (85) |
|  | in vivo: 4–6-weeks-old female C57BL/6 mice vaccinated with B16-F10 | curcumin: 50 mg/kg/d, 12 d; po  apigenin: 150 mg/kg/d, 12 d; po | + immune cell infiltration; T cell immunity  − melanoma xenograft growth; PD-L1 expression in DCs |  |
| CUR | in vitro: Cal 27 and FaDu tongue squamous cell carcinoma cell lines | 5μM, 10μM; 24 h | + CD8+ T cell number  − expression of PD-L1 and p-STAT3Y705 in cancer cells | (86) |
|  | in vivo: 4NQO mice model: 4-6 weeks-old female C57B1/6 mice, 4NQO given in drinking water for 16 weeks | 4 weeks |  |  |
| CUR | in vitro: Hep3B human hepatoma cells- peripheral blood mononuclear cells (PBMCs) co-cultivation system | 30µM; 24 h | + PBMCs proliferation; the number of CD8+ T cells; LDH levels  − the expression of PD-L1 on the surface of Hep3B cells; the number of FOXP3+ Tregs; expression of TGF-β1 | (87) |
|  | in vivo: nude mice vaccinated with Hep3B cells | -/-; ip |  |  |
| CUR | in vivo: 5-weeks-old C57BL/6J mice vaccinated with Lewis lung carcinoma cell | 50 mg/kg; 20-24 d | − the expression level of Arg-1 and ROS; accumulation of MDSCs in spleen and tumor tissues; IL-6 levels | (88) |
|  | in vitro: MDSCs and T cells co-cultivation system | 5μM, 10μM, 20μM; 12 h |  |  |
| curcuminoid | in vitro: NK cells-MP2 pancreatic cancer cells co-cultivation system | curcuminoids-omega-3-antioxidants (smartfish): 27 pg/ml; 48-96 h | + pancreatic cancer cell apoptosis; cytotoxic activity of NK cells; Capase-3  − IFN-γ production in NK cells | (90, 91) |
| CUR | in vitro: MDA-MB-231 human breast carcinoma and NK-92 cells co-cultivation system | 0, 0.1, 1, 10 μM; 5 h | + the frequency of CD16+ and CD56dim NK-92 cells; NK cell cytotoxicity; the expression of pstat4 and pstat5 proteins in NK-92 cells  − the expression of pErk and PI3K proteins in cancer cells | (93) |
| RES | in vivo: 7-weeks-old female C57BL/6 mice vaccinated with B16F10 melanoma cells | 0.5 mg/kg; days -2, 0, 2, 4, 6, and 8; iv | + cytotoxicity of NK cells；IL-2 induced IFN-γ secretion by NK cells；expression of CD107a in NK cells | (94) |
|  | in vitro: NK92 the human NK cell lines-K562 cells co-cultivation system | 20 μM; 36 h |  |  |
| RES | in vivo:subcutaneous HCC tumor models:  6-8-weeks-old C57BL/6 mice vaccinated with Hepa1-6 cells  orthotopic HCC tumor model:  6-8-weeks-old BALB/c mouse transplanted with H22 tumor mass | 50 mg/kg; 3 weeks | + TNF-α and IFN-γ; the proportion of CD8+T cells in tumor  − the proportion of CD8+CD122 Tregs and M2 TAMs in tumors; TGF-β1 and IL-10 | (95) |
| anthocyanin | in vivo: NMBA-induced esophageal papilloma mouse model: 3-5-week-old Male F-344 rats, N-nitrosomethylbenzylamine(NMBA): 0.35 mg/kg; 5 weeks; ip | AIN-76A synthetic diet supplemented with 3.8 μmol/g; 15/25/35 weeks | + inflammatory macrophages, M2 TAMs and neutrophils recruitment in tumors | (96) |
| EGCG | in vitro: 4T1 breast cancer cell line | 0/50/100/150/200/250 μg/mL; 24/48 h | + the ratio of CD4+ Tregs and CD8+ T cells in tumor  − the ratio of MDSCs in tumor | (97) |
|  | in vivo: 6-week-old male BALB/c mice vaccinated with 4T1 cells | pretreatment: 250/500/1000/20000 μg/mL; 1 month; po |  |  |
| apigenin | in vivo:vectopic pancreas Cancer (HPC)/KC-HPC mice: 6-8 weeks-old female C3H/He mice vaccinated with Panc02/UN-KC-6141 cells | 25 mg/kg;3 times/week;  16-17 d;ip | + M1 TAM in tumor | (99) |
|  | in vivo: SHIPWT/ko-HPC mice: 4-6 weeks-old female C57BL/6 SHIPWT/ko mice vaccinated with Panc02 adenocarcinoma cells | 25 mg/kg;3 times/week;  14-15 d;ip | − M1 TAM in tumor |  |
|  | in vivo: orthotopic pancreas Cancer mice:6-8 weeks-old C57BL/6N mice vaccinated with Panc02 adenocarcinoma cells | 25 mg/kg; 3 times/week;16-20 d; ip | + SHIP-1 expression;M1 TAM in tumor  − inflammation;MDSC amplification |  |
| EGCG | in vitro: 4T1 breast cancer cell line |  | + the expression of TNF-α in TAMs; miR-16 in exosomes  − macrophage infiltration;expression of CSF-1 and CCL-2 in cancer cells; the expression of IL-6 and TGF-β in TAMs; NF-κB activity | (100) |
|  | in vivo: BALB/c mice vaccinated with 4T1 cells | -; 5 weeks; - |  |  |
| CUR nanomicelle  （CUR@PPC） | in vitro: B16F10 melanoma cells-RAW264.7 macrophages co-cultivation system | curcumin 10 μM; 10 h | − expression of TGF-β, IL-10 in RAW264.7; expression of CCL-22, pho-p65, PD-L1 protein in both the B16F10 and RAW264.7 cells | (102) |
|  | in vivo: C57BL/6 mice vaccinated with B16-F10 | intratumoral curcumin >10 μM; 1 time/3 day (from the sixth day); 12 day | + tumor infiltration of CD8+T cell; IFN-γ; TNF-α; GZMB  − expression of CCL-22, PD-L1, TGF-β, IL-10; tumor infiltration of Tregs |  |
| GBM-antibody-linked CCP | in vivo: 2–6 months old C57BL6 male mice vaccinated with glioblastoma GL261 cells | short-term treatment: 2 mg /d; 5d; ip | + M1; NK cell recruitment; P-STAT1 and STAT1 in TAM; IL-12; MCP-1; caspase-3; IFN-γ  − M2; P-STAT3 and STAT3 in TAM; IL-10 | (103) |
| TriCurin(CUR:EGCG:RES=4:1:12.5) | in vivo:TC-1 mouse model | 2.5 µl of the 1.28 mM +/24 h; 5 d | + M1; IL-12; NK cell and CTLs recruitment  − M2 | (105) |
| nanoscale curcumin and resveratrol | in vitro: the mice CT26 colon cancer cells | 1.0-2.5 μM | + cell cycle arrest in CT26; the expression of macrophages and T-lymphocytes and Hsp70  − the cell viability in CT26 | (106) |
|  | in vivo: 6 weeks-old female BALB/c mice vaccinated with CT26 cells | 200 μg nanoscale curcumin and 105 μg nanoscale resveratrol/2 d; 14 d |  |  |
| theracurmin | in vitro: DCs(transfection with OE19, OE33 RNA)-T cell /CTLs co-cultivation system | 50 μM; 48 h | + CD86 expression of DCs; the secretion of IFN-γ in CTLs  − the secretion of TNF-α; IL-8, IL-6, IL-10 and IL-1β in activates T cells; the secretion of IL-8 in CTLs | (107) |
| GO-Y030 | in vitro: CD4+ CD25+ T cell-hIL-2(10 ng/ml )-anti-IFN-γ (10 μg/ml) co-cultivation system  CD8+ T cells-CD4+CD25+ Tregs-Dynabeads™ T-activator CD3/CD28 co-cultivation system | 0.25 μM; 3 d | + apoptosis  − the generation of Foxp3+ Tregs; histone acetyltransferase p300 activity; Foxp3 gene expression; Treg stability; the secretion of TGF-β and IL-10 in Tregs; the activation of STAT3 | (109) |
|  | in vivo: 7-12 weeks old C57BL/6 mice vaccinated with B16-F10 melanoma cells | 5 mg/kg; 6-9 d |  |  |
| TSA | in vivo: 6-7 weeks-old wild-type inbred BALB/c mice vaccinated with Her2/CT26 colon cancer cells | 10mg/kg, 3 times/week; -; ip | − the number and ratio of CD4+CD25+FoxP3+ Treg and MDSCs in tumor | (108) |
| TPL | in vitro: platinum-resistant SKOV3/DDP epithelial ovarian cancer cell line | TPL: 8 ng/ml  TPL: 8 ng/ml+DDP: 10 µg/ml; 24 h | + NK cell-related protein levels of CD16 and CD56  − migration and invasion of cancer cells;the expression of ITG β1 and survivin, MMP-2 and MMP-9 of cancer cells | (110) |
|  | in vivo: BALB/C-nu nude mice vaccinated with SKOV3/DDP cells | TPL: 0.15 mg/kg/day  DDP: 4 mg/kg/day; the 1st and  8th days + TPL: 0.15 mg/kg/day; -; ip |  |  |
| caffeic acid phenethyl ester | in vivo: 3-week-old wildtype BALB/c mice transplanted with the Trp53–/– donor mammary fragments in the inguinal fatpads and then accepted SIR (10, 50, or 100 cGy; 3 days) or DIR (0 cGy for Fe, 49 cGy for Ar, and 30 cGy for Si; 3 days) | 0.47 mg/kg, 23.5mg/kg, 47mg/kg; 7012 meal; diet | + lymphocyte infiltration  − production of cytokines and chemokines; T cell proliferation; COX-2, PD-L1 expression; cancer aggressiveness | (111) |
| HS-1793 | in vitro:bbFM3A breast cancer cell lines | HS-1793: 0-10 µg /ml and/or 1.88 Gy/min irradiation | + the number of CD8+ T cell; IFN-γ secretion  − the number of Tregs; Tregs infiltration | (112) |
|  | in vivo:b6 weeks-old female C3H/He mice vaccinated with FM3A breast cancer cells | HS-1793: 0.5 or l mg/kg, 2 times/week; 3 weeks  irradiation: 5 Gy/min |  |  |
